# Is the PrePex device an alternative for surgical male circumcision in adolescents ages 13–17 years? Findings from routine service delivery during active surveillance in Zimbabwe

**DOI:** 10.1371/journal.pone.0213399

**Published:** 2019-03-11

**Authors:** Webster Mavhu, Karin Hatzold, Ngonidzashe Madidi, Brian Maponga, Roy Dhlamini, Malvern Munjoma, Sinokuthemba Xaba, Getrude Ncube, Owen Mugurungi, Frances M. Cowan

**Affiliations:** 1 Centre for Sexual Health and HIV/AIDS Research (CeSHHAR), Harare, Zimbabwe; 2 Liverpool School of Tropical Medicine, Liverpool, United Kingdom; 3 Population Services International (PSI), Harare, Zimbabwe; 4 Ministry of Health & Child Care, Harare, Zimbabwe; KEMRI Wellcome Trust Research Programme, KENYA

## Abstract

**Background:**

Male circumcision devices have the potential to accelerate adolescent voluntary medical male circumcision roll-out. Here, we present findings on safety, acceptability and satisfaction from active surveillance of PrePex implementation among 618 adolescent males (13–17 years) circumcised in Zimbabwe.

**Methods:**

The first 618 adolescents consecutively circumcised from October 2015 to October 2016 using PrePex during routine service delivery were actively followed up. Outcome measures included PrePex uptake, attendance for post-circumcision visits and adverse events (AEs). A survey was conducted amongst 500 consecutive active surveillance clients to assess acceptability and satisfaction with PrePex.

**Results:**

A total of 1,811 adolescent males were circumcised across the three PrePex active surveillance sites. Of these, 870 (48%) opted for PrePex but only 618/870 (71%) were eligible. Among the 618, two (0.3%) self-removals requiring surgery (severe AEs), were observed. Four (0.6%) removals by providers (moderate AEs) did not require surgery. Another 6 (1%) mild AEs were due to: bleeding (n = 2), swelling (n = 2), and infection (n = 2). All AEs resolved without sequelae. Adherence to follow-up appointments was high (97.7% attended 7 day visit). A high proportion (71.6%) of survey respondents said they heard about PrePex from a mobilizer; 49.8% said they chose PrePex because they wanted to avoid the pain associated with the surgical procedure/surgery on their penis. Acceptability and satisfaction with PrePex was high; 95.4% indicated willingness to recommend PrePex to peers. A majority (92%) reported experiencing pain when PrePex was being removed.

**Conclusions:**

Active surveillance of the first 618 adolescent males circumcised using PrePex suggests that the device is both safe and acceptable when used in routine service delivery among 13–17 year-olds. There is need to intensify specific demand generation activities for PrePex male circumcision among this group of males.

## Introduction

Over the last 10 years, at least 14 African countries have implemented voluntary medical male circumcision (VMMC) for HIV prevention [1, 2]. By December 2017, a cumulative total of 18.6 million VMMCs (representing 90% of the global target of 20.8 million set in 2011) had been performed in these countries [2]. The 18.6 million VMMCs had already averted an estimated 230,000 new HIV infections by 2017 and this number is projected to grow to 1.1 million by 2030 [2]. An important innovation in the delivery of VMMC has been the introduction of medical devices for adolescent and adult male circumcision (MC) [3]. To date, two devices, PrePex and ShangRing, have been prequalified by World Health Organization (WHO) and can be used in VMMC programs supported by the President’s Emergency Plan for AIDS Relief (PEPFAR) and the Global Fund for AIDS, Tuberculosis and Malaria [3, 4].

Medical devices for MC have the potential to accelerate VMMC roll-out by making the procedure easier, quicker and more widely accessible [5, 6]. Additionally, choice is an important factor in enhancing acceptability and uptake of health services [3]. VMMC devices provide an alternative for males who are hesitant to undergo conventional surgery. During active surveillance of the PrePex device among adult males in Zimbabwe (conducted 2014), out of 2,156 men offered VMMC, 46.4% chose PrePex over conventional surgery [7], with some citing the desire to avoid surgery on their penis as the main reason for choosing the device.

PrePex (a device which works by compressing the foreskin between a ring and an elastic band, leading to distal tissue necrosis) [8] received WHO full prequalification in August 2016 after being assessed as set out in WHO *Framework for Clinical Evaluation of Male Circumcision Device*s [9]. Following results from studies conducted in Rwanda [10, 11], additional research with the device was conducted in Zimbabwe to establish its safety, efficacy and acceptability among providers and clients [12–14]. Data from these studies contributed to the prequalification of PrePex by WHO for use in adults aged ≥18 years [15]. In addition to the criteria defined in the *Framework for Clinical Evaluation of Devices for Male Circumcision* [9], WHO also outlined an evaluation series that each country considering introduction of medical devices for MC should complete.

The WHO Technical Advisory Group (TAG) on Innovations in Male Circumcision recommended active follow-up of the first 1,000 clients circumcised using a new device after prequalification, and undertaken within the context of routine service delivery [16]. The main purpose of the active follow-up (surveillance) is to assess safety through capturing, among these 1,000 clients, all complications and adverse events under field (rather than research) conditions. Zimbabwe was among the first countries to actively follow up 1,000 clients ≥18 years circumcised using PrePex during routine service delivery [7].

In late 2014, the WHO TAG on Innovations in Male Circumcision reviewed data on safety and acceptability of PrePex in adolescents that were available from a bridging study involving successful placements in 402 adolescents (13–17 years) in Zimbabwe [17]. The TAG also reviewed partial findings from another PrePex study conducted in South Africa, which included 89 males 13–17 years [18]. Based on findings from the two countries, the TAG recommended that PrePex use could be extended to include eligible adolescents 13–17 years, but only under active surveillance since the numbers assessed were small but within the range noted in the WHO clinical evaluation framework for a bridging study [19]. The TAG subsequently advised that active surveillance of adolescent PrePex VMMC be undertaken [19].

Following the TAG recommendation, Zimbabwe actively followed up adolescents 13–17 years circumcised using PrePex during routine service delivery, where the device was marketed through various channels including mass media (radio, television); small media (newspaper/brochure) and; interpersonal communication (VMMC mobilizer, health-care worker). Of note, prior to undergoing MC, all males must be screened for medical eligibility, in particular the absence of any penile abnormalities and current genital infections [16]. With the PrePex device, use may be further restricted due to additional anatomical reasons (e.g. phimosis—inability to retract foreskin), a narrow foreskin opening or a short frenulum; or technical reasons that preclude device placement (e.g. unavailability of a correct device size) [16].

Here, we present findings on acceptability, safety and satisfaction among all males 13–17 years circumcised using PrePex during active surveillance between October 2015 and October 2016.

## Methods

### Overview

Data presented here are from i) active surveillance of male adolescents consecutively circumcised using PrePex during routine service delivery between October 2015 and October 2016 and ii) a survey conducted amongst 500 consecutive active surveillance clients to assess acceptability and satisfaction with PrePex.

### Active surveillance

Between 19 October 2015 and 31 October 2016, PrePex circumcisions were conducted at three VMMC clinics in Zimbabwe’s two largest provinces (Harare n = 1 clinic, Bulawayo n = 2 clinics). The three clinics had previously been sites for adult PrePex active surveillance [7]. VMMC staff at the three sites received refresher training on active surveillance standard operating procedures (SOP) and data collection tools. In addition, male researchers were deployed at the three sites specifically for data collection and client follow up.

### Outcome measures

Outcome measures for the active surveillance included i) percentage of male adolescents seeking VMMC who opted for circumcision by PrePex rather than surgery, ii) percentage of PrePex clients failing to return to the clinic for scheduled follow up appointment on days 7, 14 and 49, iii) percentage of PrePex clients returning to the clinic for each scheduled appointment after receiving reminders and iv) percentage of adverse events (AEs). AEs were classified according to the surveillance SOP and PrePex AE guidelines. Of note, early removals (i.e. within days 1–6 of placement) requiring surgery were classified as severe AEs, the rest were recorded as moderate AEs. Pain was assessed using a visual analogue score with possible values of 0, 20, 40, 60, 80, and 100 where 0 corresponded to "no pain at all" and 100 to "most severe pain" and measured at two time points—while wearing PrePex and at removal.

### Client active follow-up

During active surveillance, all male adolescents (13–17 years) seeking VMMC at the three clinics between October 2015 and October 2016 who opted and were eligible for circumcision using PrePex, were actively followed up for their PrePex circumcision and post-op wound care. Clients provided their mobile phone numbers and home addresses so they could be tracked in the event that they missed a scheduled post-circumcision appointment. In addition, clients were instructed to return to the clinic outside scheduled appointments if they experienced any AEs or complications with the device.

For active surveillance, if a client failed to attend for their appointment seven days post circumcision (day 7), clinic staff made at least three attempts to contact him by phone and made at least two home visits, if necessary. If a client failed to attend a scheduled appointment after removal of the device (days 14 or 49), clinic staff made at least three attempts to contact him by phone but no home visits were conducted. All missed appointments were rescheduled to a time that was convenient to the client and consistent with clinic hours. If the client was unable to attend, he was assessed over the phone using a standard set of questions to determine the presence of AEs, the extent of wound healing and any wound care practices. AEs were documented at each appointment. All attempts to contact the client were recorded on a contact log as were reasons reported for missing the scheduled appointment. Routine VMMC monitoring data as per national guidelines were also collected.

### Satisfaction survey

Five hundred consecutive active surveillance male adolescents who had undergone PrePex male circumcision, attending for their scheduled appointment on day 14, were asked to take a short interviewer-administered structured questionnaire. The questionnaire included questions from other PrePex acceptability and satisfaction studies [7, 20]. It was developed in English and translated into Shona and Ndebele, Zimbabwe’s dominant indigenous languages, also spoken and understood by smaller ethnic groups. It was then pretested with twenty 13–17 year-olds and subsequently refined based on their feedback. Questionnaire items explored among others issues, reasons for choosing PrePex, satisfaction with the procedure as well as perceptions of pain, odor and (in)convenience (S1 File).

Survey respondents were asked to indicate pain severity on a numerical scale ranging from 0 (no pain) to 100 (most severe pain). To enhance comparability of results, a pain score of at least 60 was considered severe in line with a previous acceptability and satisfaction study among adult men [7]. Survey respondents were also asked to indicate discomfort with odor on a numerical scale ranging from 0 (no odor) to 100 (strongest odor). Additionally, they were asked to indicate satisfaction with PrePex circumcision outcome on a numerical scale ranging from 0 (no satisfaction) to 100 (highest satisfaction). The questionnaire was programmed using Entryware software and tablets were used for data collection. Skip instructions and mandatory data fields were used to ensure data validity, consistency and completeness. The questionnaire was administered by trained male researchers in either Shona or Ndebele.

### Data processing and analysis

Active surveillance data from the three sites were entered into a database and analyzed to ascertain the percentage of male adolescents seeking VMMC who chose PrePex over the surgical procedure, the percentage of PrePex clients failing to return to clinic (days 7, 14 and 49), the percentage of PrePex clients returning to the clinic for each scheduled appointment after receiving reminders and, percentage of AEs.

Questionnaire data were downloaded into an Access database. Completeness and consistency checks were performed. Any anomalies in the data were verified and corrected. Descriptive analyses of key variables were performed. Data were analyzed using Stata 14. A chi-squared test assessed association between pain at device removal and likelihood of recommending PrePex to others; a p-value of <0.05 was considered significant.

### Ethical considerations

This acceptability and satisfaction study (plus active surveillance/secondary data use) was approved by the Medical Research Council of Zimbabwe (A/1810) and the University College London ethics committee (2538/003). All acceptability and satisfaction study participants provided written informed assent in addition to caregiver consent.

## Results

### Active surveillance: PrePex preference, eligibility and uptake

Between 19 October 2015 and 31 October 2016, a total of 1,811 male adolescents (13–17 years) were circumcised across the three PrePex active surveillance sites. Of these, 870 (48%) opted for PrePex but only 618/870 (71%) were eligible. Reasons for PrePex ineligibility were adhesions/tight foreskin (n = 134/252, 53.2%), mostly in 13 and 14 year-olds (n = 125/134, 93.3%); presence of sexually transmitted infections (n = 5/252, 2%), all in 15–17 year-olds; urinary tract infection (n = 2/252, 0.8) and biological penile anomalies (n = 12/252, 4.8%). In 99/252 adolescents (39.3%), the available PrePex device sizes were too large, mostly in 13 and 14 year olds (n = 86/99, 86.9%); the remainder (n = 13/99, 13.1%) were 15–16 year olds.

### Active surveillance: Frequency and outcomes of follow-up

There was good adherence to follow-up appointments with 604 (97.7%) clients returning to the VMMC site for their scheduled appointment on day 7 without the need for any reminder. All who did not return to the VMMC site for their day 7 appointment (n = 14, 2.3%) were successfully tracked. Of these, 13 (92.9%) returned to the VMMC clinic on day 8 after at least 2 text message reminders and 2 call attempts. All cited school commitment as their reason for missing the scheduled appointment. The remaining client (7.1%) returned on day 9 (Monday) after missing a pickup vehicle deployed to help circumcised males attend for follow up on day 7 (Friday). A total of 112 (18.1%) adolescents circumcised using PrePex failed to attend their scheduled appointment on day 14, and over two thirds 423 (68.4%) failed to attend for review on day 49. All that missed the day 49 review but were later reached by phone (n = 417, 98.6%) reported that complete healing had been achieved.

### Safety: Adverse events

Table 1 summarizes AEs that occurred during active surveillance. A total of 12/618 (1.9%) AEs were observed, of which six (1%) were classified as either moderate (n = 4) or severe (n = 2), and six (1%) as mild, as per the active surveillance SOP. The two (0.3%) severe AEs were self-removals and required surgery (severe AEs). In both cases, adolescents removed the device themselves on day 2 due to pain and dorsal slit circumcision was perfomed 24 hours post self-removal. The four (0.6%) moderate AEs were early removals by providers—because the clients complained of pain (moderate AEs)—that did not require surgery. They occurred on days 5 (n = 3) and 6 (n = 1). In all cases, the foreskin had already necrotized and was easy to remove. Mild AEs were due to: bleeding (n = 2), swelling (n = 2), and infection (n = 2). All AEs resolved without sequelae.

**Table 1:** Number, nature and severity of PrePex VMMC AEs (N = 12).

No. (%)	Nature	Severity
6 (1%)	Bleeding (n = 2	Mild
	Swelling (n = 2)	
	Infection (n = 2)	
4 (0.6%)	Early removals by clinical staff (n = 3 day 5, n = 1 day 6) due to pain (no surgery required as foreskin had already necrotized)	Moderate
2 (0.3%)	Self-removals on day 2 (n = 2) due to pain (Both were classified as severe AEs because they required surgery).	Severe

### Survey: Source of information about PrePex

Between October 2015 and August 2016, 500 adolescents who had undergone PrePex male circumcision completed an interviewer-administered questionnaire when they attended the day 14 review appointment. None among all who attended during this period declined to take part in the study. Table 2 summarizes sources of information about PrePex (also known as the "ring"). Most of the respondents (71.6%) said they heard about PrePex from a VMMC mobilizer. The most-cited sources of information about PrePex were VMMC mobilizer (71.6%), acquaintance (50.4%) and radio/television (29.6%).

**Table 2:** Sources of information about PrePex (N = 500).

Where respondent heard about "ring"	Yes (%)	No (%)
VMMC mobilizer	358 (71.6%)	142 (28.4%)
Friend/peer/parent/teacher	252 (50.4%)	248 (49.6%)
Radio/television	148 (29.6%)	352 (70.4%)
Health-care worker at VMMC site	78 (15.6%)	422 (84.4%)
Newspaper/brochure	76 (15.2%)	424 (84.8%)
Health-care worker (not at VMMC site)	15 (3%)	485 (97%)
Other	35 (7%)	465 (93%)

### Survey: Main reasons for choosing PrePex

Table 3 summarizes the main reasons for choosing PrePex. More than half of the respondents (62.2%) said they chose PrePex because they wanted to avoid the pain associated with the surgical procedure/surgery on their penis. Slightly over a quarter (26.8%) said it was so they could continue with daily activities (including school and work). About 5% chose the device because someone recommended/suggested it to them.

**Table 3:** Main reasons for choosing PrePex ("ring")—N = 500.

Main reason	N (%)
To avoid the pain associated with the surgical procedure/surgery on my penis	311 (62.2%)
So I could continue with my daily activities (including school and work)	134 (26.8%)
Someone who was circumcised with the ring recommended it to me	13 (2.6%)
My friends/peers/parents/teachers said I should try the ring	11 (2.2%)
Other	31 (6.2%)

### Survey: Acceptability and satisfaction with PrePex

Satisfaction with device was high with 487 (97.4%) respondents stating that they were satisfied/very satisfied with PrePex outcome. Device acceptability was also high with 477 adolescents (95.4%) indicating that they were somewhat likely/very likely to recommend PrePex to peers (Table 4). Dissatisfaction was due to penile swelling after removal and transient discoloration of the inner foreskin.

**Table 4:** Satisfaction with outcome and likelihood to recommend to peers (N = 500).

**Satisfaction with outcome**	**N (%)**
Very satisfied	273 (54.6%)
Satisfied	214 (42.8%)
Dissatisfied	3 (0.6%)
Very dissatisfied	10 (2%)
**Likelihood to recommend PrePex to peers**	
Very likely	370 (74%)
Somewhat likely	107 (21.4%)
A little likely	22 (4.4%)
Not at all likely	1 (0.2%)

### Survey: Perceptions of pain

A total of 425 (85%) respondents reported experiencing pain whilst wearing the device (days 2–7), with 108/425 (25.4%) estimating their pain severity to be 60–100%. More than half (n = 249/425, 58.6%) reported experiencing pain on first two days of wearing the ring, 22.8% days 3–4 and 18.6% days 5–7. Furthermore, 460 (92%) respondents reported experiencing pain when the device was being removed, with 155/460 (33.7%) estimating their pain severity to be 60–100%. Of the 460 respondents, 71 (15.4%) stated that they would have opted for surgical circumcision instead of PrePex if they had known the extent of pain. Sixty respondents (13%) stated that they would have decided not to be circumcised at all (Table 5). Nearly two-thirds (64.6%) said they did not wish anything.

**Table 5:** What participants wished due to pain during device removal (N = 460).

As a result of the pain during ring removal, did you ever wish?	N (%)
You had chosen to be circumcised surgically instead of using the ring	71 (15.4%)
You had not been circumcised at all	60 (13%)
I did not wish anything	297 (64.6%)
Other	27 (5.9%)
I did not experience pain	5 (1%)

Among 23 respondents who indicated that they were not at all likely/a little likely to recommend PrePex to their peers, 8 (34.8%) ranked their pain during device removal to be at least 60% on the numerical scale. Among 477 respondents who indicated that they were somewhat likely/very likely to recommend PrePex to peers, 147 (30.8%), (P = 0.85) ranked their pain similarly at 60% or above.

## Discussion

We present findings from active surveillance conducted among male adolescents circumcised using the PrePex device between October 2015 and October 2016 (N = 618) during routine service delivery in Zimbabwe. Two (0.3%) self-removals which required surgery (severe AEs), were observed. Four (0.6%) early removals by providers due to pain (moderate AEs) did not require surgery.

The low rate of severe or moderate AEs observed during active surveillance is consistent with what was observed in adolescent PrePex research studies [17, 18] and surveillance of the device among adult men [7]. Active surveillance among male adolescents suggests that PrePex can be safely scaled up with this group in routine VMMC program roll-out. Nonetheless, the relatively high ineligibility rate (mostly among 13 and 14 year-olds) highlighted the need for smaller device sizes (which are now available - http://prepex.com/prepex-newsletters). Of note, 2% were ineligible due to presence of sexually transmitted infections (STIs), highlighting the importance of offering VMMC to adolescents as part of a comprehensive sexual and reproductive health package [21–24].

All six (1%) moderate/severe AEs observed during active surveillance were pain-related. The experience of pain was also reported in the survey where 425 (85%) respondents reported experiencing pain whilst wearing the device and 460 (92%) when the device was being removed. This was despite a change to the analgesic protocol for PrePex VMMC clients while wearing the device (from Paracetamol to Ibuprofen) and at device removal (application of local anesthetic cream) [7]. Other studies have also found that the PrePex procedure is characterized by pain [7, 25–29]. VMMC programs therefore need to be honest and provide accurate information about pain during pre-procedure counseling and conduct active pain management as appropriate. This is particularly important since pain may result in self-removals, likely leading to surgery. Concerning self-removals, client education needs to clearly articulate that once placed, the device must remain in situ for seven days and if one desires to remove it, he must return to the clinic where it may be removed with or without surgery [4]. Most importantly, the risks of removing the device at home should be emphasized. However, since some early removals due to pain occur when the foreskin has already necrotized, VMMC programs may need to offer clients the option of earlier device removal (i.e. on days 5 and 6).

Preference for PrePex during active surveillance (48%) was lower than anticipated and lower than that of conventional surgery (52%). It had been assumed that extending the eligibility criteria to include adolescents aged 13–17 years would increase PrePex uptake [7, 30]. Given devices' potential to accelerate VMMC roll-out by making the procedure easier, quicker and more widely accessible [5, 6], our findings highlight the need to intensify specific demand generation activities for PrePex male circumcision [6]. Demand creation will need to build on the perceived comparative advantages of PrePex over conventional surgery and use these to better sell the device.

Despite high levels of self-reported pain, acceptability and satisfaction with the device was high among those clients who opted for it, with 95% of survey respondents indicating that they would recommend the device to their peers, and 97% reporting satisfaction with procedure outcome. These findings are consistent with what was observed in adolescent PrePex research studies [17, 18]. Ensuring that AEs, including those related to pain are accurately communicated and managed will likely maintain these high levels of satisfaction. Of note, a higher risk of tetanus following circumcision with PrePex compared with other circumcision methods has been noted, and WHO has shared further recommendations for device use and tetanus vaccination status prior to device-enabled VMMC [31]. Moreover, post-operative wound infection remains the most common post-procedure AE, particularly among adolescents [32–36], likely due to poor post-VMMC client wound-management [31, 36–38]. The need for interventions to enhance post-VMMC wound-care can never be overemphasized. Such interventions would give clear instructions on genital hygiene and wound-care, emphasize the benefits of prophylactic tetanus vaccination, and underscore the dangers of applying traditional medicines and substances to wounds [31, 38]. While the recommendation to restrict PrePex to individuals sufficiently vaccinated against tetanus has tremendously reduced the number of PrePex-enabled procedures [39], there might be opportunities for the device to be introduced in school-based VMMC programs in combination with accompanying vaccination initiatives.

As with surveillance among adult men [7], there was high adherence (98%) to the scheduled day 7 appointment. Similar follow-up rates to adults were observed on day 14 (82% vs. 80) with lower follow up at day 49 (32% vs. 50%)–despite text message reminders and call attempts. The sub-optimal attendance on day 49 is consistent with previous observations [40]. Among adolescents, this may also be explained by reliance on program pickup vehicles plus the inability to meet the transportation costs involved (at least 1US$ each round trip). Indeed, transportation costs have been reported as the largest out-of-pocket expenditure incurred by VMMC clients [41]. Given the sub-optimal adherence to the day 49 follow-up appointment, the absence of any AEs during this period, and subsequent assurances by clients reached by phone that complete healing had been achieved, reviewing the post-circumcision follow-up protocol will not only reduce the number of scheduled appointments but associated expenses as well. Importantly, programs should continue to offer the minimum package of VMMC at each visit including HIV testing, HIV prevention counseling, screening/treatment for STIs, condom promotion, and the VMMC procedure [21–24].

Finally, participants indicated that they had mostly learned of PrePex from mobilizers and acquaintances, suggesting the effectiveness of interpersonal communication in creating demand for VMMC in general and PrePex-led VMMC, in particular. The success of these participatory approaches in motivating males of all ages to take up VMMC has been reported in other regional settings [42]. These approaches therefore need to be intensified if fast track targets [43] are to be achieved. Of note, only a few had learned of PrePex from health-care workers, reflecting the well-recognized males' general avoidance of the formal health system [44]. Indeed, VMMC is one of the few entry points through which health services can reach male adolescents [45].

### Limitations

A potential limitation of the findings presented here is that only the males who returned for the day 14 visit were interviewed and therefore, they may not be representative of the entire population that was circumcised using PrePex. Additionally, this active surveillance was limited to 618 adolescents. We had anticipated that surveillance would include 1,000 adolescents and that this number of PrePex circumcisions would have been performed within <12 months. However, uptake was much slower than anticipated and we did not have resources to continue active surveillance beyond 12 months. In addition, WHO had gathered sufficient evidence to recommend that use of the PrePex device could be extended to eligible adolescents 13–17 years in August 2016 [4], making it unnecessary to continue in surveillance. This is, however, one of the first initiatives to actively follow up male adolescents circumcised using PrePex during routine service delivery. Findings will likely inform VMMC programs as they scale up device-led adolescent VMMC in general and adolescent PrePex VMMC, specifically.

## Conclusions

We successfully followed up the first 618 male adolescents circumcised using PrePex during routine service delivery, and surveyed 500 of them to determine device safety as well as acceptability and satisfaction. Despite high levels of self-reported pain, we found that PrePex is both safe and acceptable when used in routine service delivery among male adolescents (13–17 years). Findings highlight the need to intensify specific demand generation activities for PrePex male circumcision among this group of males. Recently-introduced smaller device sizes will partly address the high ineligibility observed so far. Lastly, these data suggest that it may not be necessary to continue to advise males to return to the clinic 49 days after they have been circumcised.

## Supporting information

S1 File Study questionnaire. (ZIP)
