# Supplementary material for: Is the PrePex device an alternative for surgical male circumcision in adolescents ages 13–17 years? Findings from routine service delivery during active surveillance in Zimbabwe
Source: PLoS One. 2019 Mar 11;14(3):e0213399. doi: 10.1371/journal.pone.0213399 (PMC6411138; doi:10.1371/journal.pone.0213399)
Supplement: S1 File — (ZIP) [file pone.0213399.s001.zip › Questionnaire/clients questionnaire_eng_v5.doc]

**Study ID. Note that this questionnaire will be completed on a mobile ring (tablet)**

1. What are the reasons you chose to be circumcised with the ring?

*Multiple responses allowed. Interviewer, do read responses*

- To avoid surgery on my penis
- To avoid the pain associated with the surgical procedure
- So I could continue with daily activities (including school and work)
- Someone who was circumcised with the ring recommended it to me
- My friends/peers/parents/teachers said I should try the ring
- Other (please specify)

2. Which was the most important reason?

*Put an X in* ***ONE*** *box only*

- To avoid surgery on my penis
- To avoid the pain associated with the surgical procedure
- So I could continue with daily activities (including school and work)
- Someone who was circumcised with the ring recommended it to me
- My friends/peers/parents/teachers said I should try the ring
- Other (please specify)

3. Where did you hear about the ring?

*Multiple responses allowed. Interviewer, do read responses*

- Radio/television
- Newspaper/brochure
- Friend/peer/parent/teacher
- VMMC mobilizer
- Health-care worker at VMMC site
- Health-care worker (not at VMMC site)
- Other (please specify)

4. Did you experience pain when you were wearing the ring?

*Put an X in one box only*

- Yes (go to 5)
- No (go to 8)

5. When did you experience pain?

*Multiple responses allowed. Interviewer, do read responses*

- While wearing the ring (first 2 days)
- While wearing the ring (days 3-4)
- While wearing the ring (days 5-7)

6. How severe was the pain while you were wearing the ring?

*Indicate degree of severity on the scale that you have been given with 10 being the worst pain imaginable and 0 being no pain at all*

7. As a result of the pain while you were wearing the ring, did you ever wish?

*Put an X in one box only*

- You had chosen to be circumcised surgically instead of using the ring
- You had not been circumcised at all
- Other (please specify)
- I did not experience pain

8. Did you experience pain when the ring was being removed?

*Put an X in one box only*

- Yes (go to 9)
- No (go to 11)

9. How severe was the pain at ring removal?

*Indicate degree of severity on the scale that you have been given with 10 being the worst pain imaginable and 0 being no pain at all*

10. As a result of the pain during ring removal, did you ever wish?

*Put an X in one box only*

- You had chosen to be circumcised surgically instead of using the ring
- You had not been circumcised at all
- Other (please specify)
- I did not experience pain

11. Did you experience any unpleasant odour at any point?

*Put an X in one box only*

- Yes (go to 12)
- No (go to 18)

12. When did you experience odour?

*Multiple responses allowed. Interviewer, do read responses*

- While wearing (first 2 days)
- While wearing (days 3-4)
- While wearing (days 5-7)
- During removal (day 7)
- After removal (days 7-10)
- After removal (after day 10)

13. To what extent did the odour make you feel uncomfortable?

*Indicate degree of discomfort on the scale that you have been given with 10 being the*

*highest level of discomfort and 0 being no discomfort at all*

14. As a result of the odour, did you ever wish?

*Put an X in one box only*

- You had chosen to be circumcised surgically instead of using the ring
- You had not been circumcised at all
- Other (please specify)

15. Did you do anything to reduce/eradicate the odour?

*Put an X in one box only*

- Yes (go to 16)
- No (go to 17)

16. What did you do?

*Please write your response in the space provided*

17. How likely is it that other people sensed the odour?

*Put an X in one box only*

- Not at all likely
- A little likely
- Somewhat likely
- Very likely

18. To what extent did wearing the ring affect your daily life?

*Indicate extent on the scale that you have been given with 10 being affected a lot and 0*

*being did not affect at all*

19. To what extent did wearing the ring make it difficult for you to pass urine?

*Indicate extent on the scale that you have been given with 10 being most difficult and 0*

*being not difficult at all*

20. To what extent did wearing the ring make it difficult for you to clean your penis?

*Indicate extent on the scale that you have been given with 10 being most difficult and 0*

*being not difficult at all*

21. To what extent did you find it difficult to care for your wound after removal of the ring?

*Indicate extent on the scale that you have been given with 10 being most difficult and 0*

*being not difficult at all*

22. What issues/topics do you wish had been discussed more during the counselling process?

*Multiple responses allowed. Interviewer, do read responses*

- Nothing
- The application of the ring
- The removal of the ring
- The need to abstain from sex
- Pain
- Odour
- How to keep the penis clean while wearing the ring
- Wound care
- Other (please specify)
- Don't know

23. Overall, how satisfied or dissatisfied are you with circumcision using the ring?

*Put an X in one box only*

- Very dissatisfied
- Dissatisfied
- Satisfied
- Very satisfied

24. How likely are you to recommend that other males your age get circumcised with the ring?

*Put an X in one box only*

- Not at all likely
- A little likely
- Somewhat likely
- Very likely

**Appendix A: Scale to be used to indicate severity/extent of several issues being explored.**
