# Supplementary material for: Is the PrePex device an alternative for surgical male circumcision in adolescents ages 13–17 years? Findings from routine service delivery during active surveillance in Zimbabwe
Source: PLoS One. 2019 Mar 11;14(3):e0213399. doi: 10.1371/journal.pone.0213399 (PMC6411138; doi:10.1371/journal.pone.0213399)
Supplement: S1 File — (ZIP) [file pone.0213399.s001.zip › Questionnaire/clients questionnaire_ndeb_v5.doc]

**Study ID. Note that this questionnaire will be completed on a mobile device (tablet)**

1. **Yiziphi izizatho ezenza ukhethe ukusokwa ngeringi?**

*Impendulo ezimbalwa ziyavunyelwa. Umqhubi wengxoxo kumele abale impendulo*

- Ukubalekela ukusikwa esithweni sangensitha
- Ukubalekela ubuhlungu obuhamba lokusokwa nge-ophareshini
- Ukuze ngenelise ukuqhubeka ngisenza imisebenzi yami (kugoqela isikolo lomsebenzi)
- Omunye owasokwa ngeringi wangikhuthaza ukuthi ngikhethe yona
- Abangane/ontanga/abazali/ababalisi bathi kumele ngizame iringi.
- Ezinye (siyacela uchasise)

2. Yisiphi isizatho esasiqakatheke kulazo zonke?

*Faka u X ebhokisini* ***ELILODWA*** *kuphela*

- *Ukubalekela ukusikwa esithweni sangensitha*
- *Ukubalekela ubuhlungu obuhamba lokusokwa nge-ophareshini*
- *Ukuze ngenelise ukuqhubeka ngisenza imisebenzi yami (kugoqela isikolo lomsebenzi)*
- *Omunye owasokwa ngeringi wangikhuthaza ukuthi ngikhethe yona*
- *Abangane/ontanga/abazali/ababalisi bathi kumele ngizame iringi.*
- *Ezinye (siyacela uchasise)*

3. Iringi wayizwela nga?

*Impendulo ezimbalwa ziyavunyelwa. Umqhubi wengxoxo kumele abale impendulo*

- Erediyweni/Ku-TV
- Kuphephandaba/egwadlaneni elilemibiko ethile ebizwa ngokuthiwa yi-*brochure*
- Kumngane/ntanga/mzali/mbalisi
- Kulabo abakhankasela ukuthi abesilisa basoke ngokuzithandela endaweni zempilakahle
- Kowezempilakahle owayesendaweni lapho okusokwa khona abesilisa ngokuzithandela

kulandelwa izimiso zabezempilakahle

- Kowezempilakahle owayengekho lapho okusokwa khona abesilisa ngokuzithandela

kulandelwa izimiso zabezempilakahle

- Ezinye (siyacela uchasise)

4. Uke wezwa ubuhlungu ngesikhathi ugqoke iring?

*Faka u X ebhokisini* ***ELILODWA*** *kuphela*

- Yebo (Yana ku- 5)
- Hatshi (Yana ku-8)

5. Wabuzwa nini ubuhlungu?

*Impendulo ezimbalwa ziyavunyelwa. Umqhubi wengxoxo kumele abale impendulo*

- Ngesikhathi ngigqoke iringi (amalanga akuqala amabili)
- Ngesikhathi ngigqoke iringi (ilanga lesithathu kusiya kwelesine)
- Ngesikhathi ngigqoke iringi (ilanga lesihlanu kusiya kwelesikhombisa)

6. Kwakubuhlungu okunganani ngesikhathi ugqoke iringi?

*Tshengisa isilinganiso sebanga lobuhlungu esikalini osinikiweyo ngaphansi. Ibanga lika-100 likhomba ubuhlungu obukhulu kakhulu ongabucabanga kukanti ibanga lika-0 likhomba ukungezwa ubuhlungu ngitsho lakancane.*

7. Ngenxa yobuhlungu owabuzwayo, wake wafisa ukuthi?

*Bhala uX ebhokisini elilodwa kuphela*

- Ngabe wakhetha ukusokwa nge-ophareshini kulokuba usebenzise iringi
- Ngabe vele awusokwanga
- Okunye (*Sicela uchasise)*
- Angizwanga ubuhlungu

8. Wezwa ubuhlungu na ngesikhathi iringi ikhutshwa?

*Faka u X ebhokisini* ***ELILODWA*** *kuphela*

- Yebo (yana ku- 9)
- Hatshi (yana ku-11)

9. Ubuhlungu babungakanani ngesikhathi iringi ikhutshwa?

*Tshengisa isilinganiso sebanga lobuhlungu esikalini osinikiweyo ngaphansi. Ibanga lika-100 likhomba ubuhlungu obukhulu kakhulu ongabucabanga kukanti ibanga lika-0 likhomba ukungezwa ubuhlungu ngitsho lakancane.*

10. Ngenxa yobuhlungu owabuzwayo ngesikhathi iringi ikhutshwa wake wafisa?

*Bhala uX ebhokisini elilodwa kuphela*

- Ngabe wakhetha ukusokwa nge-ophareshini kulokuba usebenzise iringi
- Ngabe vele awusokwanga
- Okunye (*Sicela uchasise)*
- Angizwanga ubuhlungu

11. Uke wezwa umnuko ongawuthandanga ngaloba siphi isikhathi nje?

*Bhala uX ebhokisini elilodwa kuphela*

- Yebo (yana ku-12)
- Hatshi (yana ku-18)

12. Wawuzwa nini umnuko lowu?

*Impendulo ezimbalwa ziyavunyelwa. Umqhubi wengxoxo kumele abale impendulo*

- Ngesikhathi ngigqoke iringi (amalanga akuqala amabili)
- Ngesikhathi ngigqoke iringi (ilanga lesithathu kusiya kwelesine)
- Ngesikhathi ngigqoke iringi (ilanga lesihlanu kusiya kwelesikhombisa)
- Ngesikhathi kukhutshwa iringi (ilanga lesikhombisa)
- Ngemva kokukhutshwa iringi (ilanga lesikhombisa kusiya kweletshumi)
- Ngemva kokukhutshwa iringi (sekudlule ilanga letshumi)

13. Umnuko lowu ubukwenza ukuthi ungahlaliseki okungakanani?

*Tshengisa esikalini osinikiweyo ibanga lokungahlaliseki. Ibanga 100 likhombisa izinga eliphezulu kulawo wonke ngokungahlaliseki kukanti ibanga 0 litsho ukuthi akuzange kube lokungahlaliseki ngitsho lakancane*

14. **Ngenxa yomnuko lowu uke wafisa ukuthi**

*Faka uX ebhokisini elilodwa kuphela*

- Ngabe wakhetha ukusokwa ngendlela ye-ophareshini hatshi iringi
- Ngabe vele awusokwanga
- Okunye (*Sicela uchasise)*

15. **Kukhona owakwenzayo yini ukuze wehlise kumbe uqede umnuko lowu?**

*Faka uX ebhokisini elilodwa kuphela*

- Yebo (hamba ku-16)
- Hatshi (hamba ku-17)

16. Wenzani?

*Sicela ubhale phansi emkhandlweni owuphiweyo*

17. Kulethuba elinganani lokuthi abanye abantu bawuzwa umnuko lo?

*Faka u X ebhokisini elilodwa kuphela*

- Alikho lelo thuba
- Kulethuba elincane
- Likhonanyana ithuba
- Kulethuba elikhulu

18. Ukugqoka iringi kwaphambanisa kangakanani impilo yakho yansukuzonke?

*Tshengisa esikalini osinikiweyo ibanga lokungahlaliseki. Ibanga 100 likhombisa izinga eliphezulu lokuphambaniseka kwempilo yakho kukanti ibanga 0 litsho ukuthi impilo yakho kayizange iphambaniseke lakancane*

19. Ukugqoka iringi kwenza ukuthi kubenzima ukuchema ngokwebanga elingakanani?

*Tshengisa esikalini osinikiweyo ibanga lobunzima. Ibanga 100 likhombisa izinga eliphezulu lobunzima kukanti ibanga 0 litsho ukuthi kwakungelabunzima ngitsho lakancane*

20. Ukugqoka iringi kwenza ukuthi kubenzima ukugeza isitho sakho sangasese ngokwebanga elingakanani?

*Tshengisa esikalini osinikiweyo ibanga lobunzima. Ibanga 100 likhombisa izinga eliphezulu lobunzima kukanti ibanga 0 litsho ukuthi kwakungelabunzima ngitsho lakancane*

21. Wakuthola kunzima kangakanani ukunakelela isilonda sakho ngemva kokukhutshwa kweringi?

*Tshengisa esikalini osinikiweyo ibanga lobunzima. Ibanga 100 likhombisa izinga eliphezulu lobunzima kukanti ibanga 0 litsho ukuthi kwakungelabunzima ngitsho lakancane*

22. Yiziphi izindaba/izihloko obufisa ukuthi nga zathinthwa kabanzi ngesikhathi sengxoxo yokuxwayiswa?

*Impendulo ezimbalwa ziyavunyelwa. Umqhubi wengxoxo kumele abale impendulo*

- Akula
- Ukufakwa kweringi
- Ukukhutshwa kweringi
- Ukudingakala kokuzila amacansi
- Ubuhlungu
- Umnuko
- Ukuthi isitho sensitha sigcinwa njani sihlanzekile ngesikhathi iringi isagqokiwe
- Ukunakelela isilonda
- Okunye (sicela uchasise)
- Angazi

23. Sigoqela konke nje usuthiseke kumbe awusuthisekanga kangakanani ngokusokwa ngeringi?

*Faka uX ebhokisini elilodwa kuphela*

- Angisuthisekanga kakhulu
- Angisuthisekanga
- Ngisuthisekile
- Ngisuthisekile kakhulu

24. Kulethuba elingakanani lokuthi ungaxwayisa abanye abesilisa abayintanga yakho ukuthi basokwe ngeringi?

*Faka u X ebhokisini elilodwa kuphela*

- Alikho lelo thuba
- Kulethuba elincane
- Likhonanyana ithuba
- Kulethuba elikhulu

**Isengezo A: Isikali sokusetshenziswa ukutshengisa amabanga obuhlungu/ubukhulu/ubunengi/ubunzima bezinto ezimbalwa ezichwayisiswayo.**
