# Supplementary material for: Is the PrePex device an alternative for surgical male circumcision in adolescents ages 13–17 years? Findings from routine service delivery during active surveillance in Zimbabwe
Source: PLoS One. 2019 Mar 11;14(3):e0213399. doi: 10.1371/journal.pone.0213399 (PMC6411138; doi:10.1371/journal.pone.0213399)
Supplement: S1 File — (ZIP) [file pone.0213399.s001.zip › Questionnaire/clients questionnaire_sh_v5.doc]

**Study ID.**

1. Ndezvipi zvikonzero zvakaita kuti usarudze kuchecheudzwa nenzira ye*ring*?

*Mhinduro dzakawanda dzinobvumirwa. Usamuverengere mhinduro*

- Ndaisada kuchekwa panhengo yangu
- Ndaisada kurwadziwa nekuda kwekuchekwa
- Nzira iyi yaiita kuti ndikwanise kuramba ndichiita zvandaiita zuva nezuva (kusanganisira	chikoro nebasa)
- Mumwe akachecheudzwa nenzira ye*ring* akandikurudzira kuti ndisarudzewo nzira iyi
- Shamwari dzangu/vamwe vangu/vadzidzisi vangu vakati ndinofanirwa kuedza nzira iyi
- Zvimwewo (Tiudze zvikonzero zvacho)

2. Ndechipi chikonzero chainge chakanyanyokosha kwauri?

*Isa mufananidzo X mubhokisi* ***rimwe*** *chete*

- Ndaisada kuchekwa panhengo yangu
- Ndaisada kurwadziwa nekuda kwekuchekwa
- Nzira iyi yaiita kuti ndikwanise kuramba ndichiita zvandaiita zuva nezuva (kusanganisira	chikoro nebasa)
- Mumwe akachecheudzwa nenzira ye*ring* akandikurudzira kuti ndisarudzewo nzira iyi
- Shamwari dzangu/vamwe vangu/vadzidzisi vangu vakati ndinofanirwa kuedza nzira iyi
- Zvimwewo (Tiudze chikonzero chacho)

3. Ndekupi kwawakanzwa nezve*ring*?

*Mhinduro dzakawanda dzinobvumirwa. Usamuverengere mhinduro*

- Redhiyo/terevhizheni
- Pepanhau/chimwewo chinyorwa
- Shamwari/mumwewo mukomana/mubereki/mudzidzisi
- *VMMC mobilizer*
- Mushandi wehutano anoshandira panzvimbo inochecheudzirwa vanhurume
- Mushandi wehutano asingashandiri panzvimbo inochecheudzirwa vanhurume
- Kumwewo (Tiudze kunhu kwacho)

4. Wakarwadziwa here pawainge wakapfeka *ring*?

*Isa mufananidzo X mubhokisi rimwe chete*

- Hongu (enda pamubvunzo 5)
- Kwete (enda pamubvunzo 8)

5. Ndepapi pawakarwadziwa?

*Mhinduro dzakawanda dzinobvumirwa. Usamuverengere mhinduro*

- Pandainge ndakapfeka *ring* (pamazuva 2 ekutanga)
- Pandainge ndakapfeka *ring* (pamazuva 3 kusvika ku4)
- Pandainge ndakapfeka *ring* (pamazuva 5 kusvika ku7)

6. Wakarwadziwa zvakadii pawainge wakapfeka *ring*?

*Shandisa chipimo chawapihwa kuratidza kuti wakarwadziwa zvakadii. 100 anomirira kurwadziwa kwakanyanyisa uye 0 anomirira kusarwadziwa zvachose*

7. Nekuda kwekurwadziwa pawainge wakapfeka *ring*, wakashuvira here?

*Isa mufananidzo X mubhokisi rimwe chete*

- Kuti dai zvako wakachecheudzwa nenzira isiri yekushandisa *ring*
- Kuti dai zvako usina kumbochecheudzwa zvachose
- Zvimwewo (Tiudze kuti wakashuvira zvipi)
- Handina kumbobvira ndarwadziwa

8. Wakarwadziwa here pawaibviswa *ring*?

*Isa mufananidzo X mubhokisi rimwe chete*

- Hongu (enda pamubvunzo 9)
- Kwete (enda pamubvunzo 11)

9. Wakarwadziwa zvakadii pawaibviswa *ring*?

*Shandisa chipimo chawapihwa kuratidza kuti wakarwadziwa zvakadii. 100 anomirira kurwadziwa kwakanyanyisa uye 0 anomirira kusarwadziwa zvachose*

10. Nekuda kwekurwadziwa pawaibviswa *ring*, wakashuvira here?

*Isa mufananidzo X mubhokisi rimwe chete*

- Kuti dai zvako wakachecheudzwa nenzira isiri yekushandisa *ring*
- Kuti dai zvako usina kumbochecheudzwa zvachose
- Zvimwewo (Tiudze kuti wakashuvira zvipi)
- Handina kumbobvira ndarwadziwa

11. Pane here pawakambonzwa nhengo yako ichiburitsa kamweya kasingafadzi nekuda kwe *ring*?

*Isa mufananidzo X mubhokisi rimwe chete*

- Hongu (enda pamubvunzo 12)
- Kwete (enda pamubvunzo 18)

12. Ndepapi pawakanzwa kamweya aka?

*Mhinduro dzakawanda dzinobvumirwa. Usamuverengere mhinduro*

- Pandainge ndakapfeka *ring* (pamazuva 2 ekutanga)
- Pandainge ndakapfeka *ring* (pamazuva 3 kusvika ku4)
- Pandainge ndakapfeka *ring* (pamazuva 5 kusvika ku7)
- Pakubviswa *ring* (zuva rechi7)
- Mushure mekubviswa *ring* (pamazuva 7 kusvika ku10)
- Mushure mekubviswa *ring* (mazuva 10 adarika)

13. Kamweya aka kaikuita kuti usagadzikane zvakadii?

*Shandisa chipimo chawapihwa kuratidza kusagadzikana. 100 anomirira kusagadzikana zvakanyanya uye 0 anoreva kuti hapana pawakanzwa kusagadzikana.*

14. Nekuda kwekamweya aka, wakashuvira here?

*Isa mufananidzo X mubhokisi rimwe chete*

- Kuti dai zvako wakachecheudzwa nenzira isiri yekushandisa *ring*
- Kuti dai zvako usina kumbochecheudzwa zvachose
- Zvimwewo (Tiudze kuti wakashuvira zvipi)

15. Pane zvawakaita here uchida kuderedza/kupedza kamweya aka?

*Isa mufananidzo X mubhokisi rimwe chete*

- Hongu (enda pamubvunzo 16)
- Kwete (enda pamubvunzo 17)

16. Zvii zvawakaita?

*Ndapota nyora mhinduro yako pamutsara wakatarwa*

17. Zvinogoneka zvakadii kuti vamwe vanhu vakanzwa kamweya aka?

*Isa mufananidzo X mubhokisi rimwe chete*

- Hazvigoneke zvachose
- Zvingagoneke zvishoma
- Zvingangogoneke
- Zvingangogoneke zvakanyanya

18. Kupfeka *ring* kwakakukanganisa zvakadii upenyu hwako hwemazuva ese?

*Shandisa chipimo chawapihwa kuratidza kuti wakakanganiswa zvakadii. 100 anomirira kukanganiswa kwakanyanyisa uye 0 anomirira kusakanganiswa zvachose*

19. Kupfeka *ring* kwakaita kuti unetsekane zvakadii nekuita weti?

*Shandisa chipimo chawapihwa kuratidza kuti wakanetsakana zvakadii. 100 anomirira kunetsekana kwakanyanyisa uye 0 anomirira kusanetsekana zvachose*

20. Kupfeka *ring* kwakaita kuti unetsekane zvakadii pakugeza nhengo yako?

*Shandisa chipimo chawapihwa kuratidza kuti wakanetsakana zvakadii. 100 anomirira kunetsekana kwakanyanyisa uye 0 anomirira kusanetsekana zvachose*

21. Wakanetsekana zvakadii pakuchengetedza chironda chako mushure mekubviswa *ring*?

*Shandisa chipimo chawapihwa kuratidza kuti wakanetsakana zvakadii. 100 anomirira kunetsekana kwakanyanyisa uye 0 anomirira kusanetsekana zvachose*

22. Ndezvipi zvaunoshuvira kuti dai zvakataurwa nezvazvo zvakanyanya pawaitsanangurirwa/pawaidzidziswa nezvekuchecheudzwa?

*Mhinduro dzakawanda dzinobvumirwa. Usamuverengere mhinduro*

- Hapana
- Kupfekedzwa *ring*
- Kubviswa *ring*
- Nyaya yekuti ndinofanirwa kumbomira bonde
- Kurwadziwa
- Kamweya kanobuda
- Kuti ndaifanirwa kuita sei kuti nhengo yangu igare yakachena pandainge ndakapfeka *ring*
- Kuchengetedza chironda
- Zvimwewo (Tiudze zvawaida kuudzwa zvacho)
- Handizivi

23. Unogutsikana zvakadii nemaratidzikiro ava kuita nhengo yako mushure mekuchecheudzwa ne*ring*?

*Isa mufananidzo X mubhokisi rimwe chete*

- Kusagutsikana zvakanyanya
- Kusagutsikana
- Kugutsikana
- Kugutsikana zvakanyanya

24. Zvingaitike zvakadii kuti ungakurudzire vamwe vakomana vezera rako kuti vachecheudzwe nenzira ye*ring*?

*Isa mufananidzo X mubhokisi rimwe chete*

- Hazvingaitike zvachose
- Zvingangoitike zvishoma
- Zvingangoitike
- Zvingangoitike zvakanyanya
- **Appendix A: Scale to be used to indicate severity/extent of several issues being explored.**
